# Surgical Cases Treated in the Buffalo General Hospital

**Published:** 1872-10

**Authors:** C. C. F. Gay

**Affiliations:** Attending Surgeon


ART. III.—*Surgical Cases Treated at the Buffalo General Hospital by* C. C. F. GAY, M. D., Attending Surgeon.

TUMORS OF THE NECK.

Mr. L., aged twenty-two years, has two tumors, one the size of the closed hand upon the left side of the neck, another half this size upon the opposite side. They have been six months growing, and the patient says they appeared directly on the subsidence of a cold. The tumors feel hard, are moveable and supposed to be encysted. In the presence of several members of the staff the tumor of lesser size was removed. The cyst was found to contain cheesy matter. The tumor could not be enucleated, but had to be dissected out, and a portion of the cyst was allowed to remain.

Before proceeding to operate upon the larger tumor, I introduced the exploring needle, on withdrawing of which a drop of pus escaped, which revealed the fact that the tumor was but a chronic abcess. I made a deep incision entirely through the tumor, and emptied it of its purulent contents, directing the use of tents to enable the wounds to heal by the slow process of granulation.

The patient was operated upon on June 18th, and on July 18th the wounds were closed, and the patient was discharged.

REMARKS.—The diagnosis in the case of the larger tumor would have been faulty had I not made use of the exploring needle, and this fact shows the importance of a more frequent use of the needle in aiding one to make correct diagnoses, since, had the needle not been employed, the operation of dissecting out this tumor would have been an operation of some gravity, from its location in one of the triangles of the neck.

This case is of interest, since it indicates the steps of the progress of the development of some of these tumors so frequently seen in this region of the body. There was but little pus contained within the walls of the tumor, and there were no signs of inflammatory action going on. There was no pain on pressure, or without pressure. The question then arises as to the length of time pus had been deposited. There were certainly no indications of recent supurative process; therefore one would conclude that inflammatory action ante-dated the operation six months, and produced this product; that a portion of the product had been absorbed, and, with a longer lapse of time, the whole would have been absorbed, leaving a well organized tumor to have been removed either by dissection or enucleation.

### EXSECTION OF LOWER EXTREMITY OF THE RADIUS FOR NECROSIS.

Mr. T. B., aged thirty-two years, entered the Hospital June 24th. Thirteen years ago he fell, and says he sprained his wrist. Since then he has not been able to use his hand. The wrist began ulcerating last Christmas. It is at present much swollen and considerably deformed. An exploratory incision made on June 28th, reveals extensive caries, and an operation was resolved upon to be made at once. The finger introduced within the joint detected loose bone, which proved to be bones of the carpus. These were removed with about one and a half inches of the lower extremity of the radius. The arm was dressed and bound loosely upon a padded splint. The lower half of the wound was left open and cold water dressings employed.

I find the following remark in my note book, made July 18th: Wound nearly closed, but there is evidence of necrosis of ulna, which will necessitate another operation for exsection of lower extremity of ulna.

July 20th, 1872.—Mr. S., aged about thirty years, has been in Hospital and under observation for several weeks. Twelve years since he fell upon the sidewalk, striking upon the coccyx. Following this fall he had coccyodynia, remaining for an indefinite period of time, but pain on pressure at this date was referred to the first and second lumbar vertebræ. Since his injury he has been able to walk, only occasionally with the aid of crutches The greater portion of this time he has maintained the recumbent position in bed, and been quite helpless. Any unusual exertion would cause pain in the lumbar region, which appeared only to be allayed by absolute rest. Scars upon the back denote that all, or nearly all, the appliances used for counter-irritation have been employed. So that he has been a sufferer in a two-fold manner; he has suffered from pain caused by the injury and suffered also from the use of the means, ordered at various times and places, to alleviate suffering.

I learned that he is and has been for a considerable time troubled with involuntary seminal emissions.

His pulse is below one hundred and his appetite usually good. There was no obstruction in the act either of defecation or micturation, and there were no signs of paralysis.

From all the information I can obtain by interrogating his history and present state, I am convinced there is neither chronic myelitis nor meningitis; that the integrity of the spinal cord and its investing membrane is not impaired. Were it otherwise. there had been the supervention of paralysis a long time since. I was therefore able to convince myself, and attempted also to convince this patient, that recovery was possible.

The cold douche to the spinal column was accordingly ordered once daily.

For the seminal emissions, I made use of the Porte Caustique. After one application he went one month without the recurrence of an emission. This treatment was persevered in until now, after a six weeks' treatment, he is able to walk erect about the wards of the Hospital—to go up stairs and down again—for fifteen or twenty minutes at a time, without the support of crutch or cane.

